# Complications of Chiropractic Manipulation in a Patient With Von Willebrand Disease: A Clinical Case Report and Literature Review

**DOI:** 10.7759/cureus.102485

**Published:** 2026-01-28

**Authors:** Omar Fernando Rodriguez-Rodriguez, A. Olán-Rovirosa, Mario J.P. Gallegos-Alvarado, Gámez Jesús A., César Osvaldo Ruiz-Rivero

**Affiliations:** 1 Orthopaedics and Traumatology, Instituto de Seguridad y Servicios Sociales de los Trabajadores del Estado (ISSSTE) Hospital Regional Monterrey, Monterrey, MEX; 2 Orthopaedics and Traumatology, Universidad de Monterrey (UDEM), Monterrey, MEX; 3 Spine Surgery, Instituto de Seguridad y Servicios Sociales de los Trabajadores del Estado (ISSSTE) Hospital Regional Monterrey, Monterrey, MEX

**Keywords:** chiropractic therapy, iliopsoas hematoma, neurologic deficit, spinal manipulation treatment, von willebrand disease

## Abstract

Von Willebrand disease (VWD) is the most common inherited bleeding disorder and predisposes patients to hemorrhagic complications following trauma or invasive procedures. Chiropractic spinal manipulation is widely used for musculoskeletal pain; however, serious complications have been reported, particularly in patients with underlying coagulopathies. Iliopsoas hematoma with secondary femoral neuropathy is an uncommon but potentially disabling condition. We present a clinical case highlighting this rare complication following chiropractic manipulation in a patient with VWD and review the relevant literature. We describe the clinical course and follow-up of a 32-year-old female patient with known VWD who developed acute neurological deficits after chiropractic manipulation. Imaging findings were analyzed using radiographs, computed tomography (CT), and magnetic resonance imaging (MRI). Hematoma volume was calculated using the ABC/2 formula, which has been well validated and shows a high correlation with volumes calculated using planimetric techniques.

An extensive review of the literature regarding iliopsoas hematomas, chiropractic complications, and management strategies in coagulopathic patients was performed. After chiropractic manipulation, the patient developed severe lumbar and inguinal pain, followed by progressive weakness and sensory impairment of the left lower limb. Imaging revealed a large left iliopsoas hematoma measuring approximately 896 cc, causing femoral nerve compression. Management included coagulation factor replacement, pain control, and interventional radiology-guided drainage, resulting in significant hematoma reduction and neurological improvement.

At the six-month follow-up, residual neuropathy and muscle atrophy persisted, although functional recovery was evident. Patients with VWD are at high risk for severe hemorrhagic complications even after seemingly minor manipulative therapies. Chiropractic spinal manipulation may precipitate life-threatening or disabling bleeding events in this population. Early recognition, appropriate imaging, correction of the coagulopathy, and multidisciplinary management are crucial to optimize outcomes. This case highlights the importance of patient counseling, risk stratification, and caution when considering alternative therapies in individuals with inherited bleeding disorders.

## Introduction

Von Willebrand disease (VWD), first described in 1926 by Erik von Willebrand, is the most common inherited bleeding disorder [1]. Originally termed “hereditary pseudohemophilia” because of its autosomal inheritance pattern (in contrast with the X-linked pattern of hemophilia A and B), it is characterized by quantitative or qualitative abnormalities of Von Willebrand factor (VWF), leading to impaired platelet adhesion and secondary coagulation defects. 

Type 1 VWD, the most frequent form, accounts for approximately 75% of cases and is defined by factor VIII activity levels < 30%, or 30%-50% in patients with a history of bleeding episodes [2]. Type 2 is characterized by qualitative defects of vWF, subdivided into IIA, IIB, and IIN, depending on whether the defect lies in polymerization, molecular weight, or platelet binding.

The physiological role of VWF is essential in hemostasis, acting as an adhesion protein between platelets and vascular endothelium and promoting platelet aggregation, both critical in bleeding control [2]. 

Chiropractic therapy is considered a complementary or alternative medical practice, consisting of manipulations, particularly spinal manipulation, where joints are moved beyond their usual range of motion at high velocity. It is often employed for lumbar pain management [1,3]; however, complications have been described in case series [4,5,6], particularly when performed in patients with preexisting conditions, like dural tear, vertebral artery dissection, subdural hematoma, Wallenberg's syndrome, multiple disc herniation, and others.

Patients with VWD are especially vulnerable to bleeding complications following trauma, invasive procedures, or surgery. This report describes the case of a young female with VWD who developed an iliopsoas hematoma and subsequent femoral neuropathy following chiropractic manipulation, underscoring the clinical challenges in managing coagulopathic patients.

## Case presentation

A 32-year-old female patient from Galeana, Nuevo Leon, Mexico, working as a teacher, presented with progressive neurological symptoms. She has a past medical history of VWD diagnosed at six months of age, treated with oral ferrous fumarate 350 mg daily. Surgical history included right oophorectomy in 2015. There was no history of prior fractures. The patient has a history of allergy to penicillins and diclofenac. The history of blood transfusions in the past was positive due to VWD. She reports complete immunizations.

The symptoms began four days before admission, following a chiropractic session. Six hours after the procedure, she developed severe lumbar and left inguinal pain (9/10 on the Visual Analog Scale), prompting her to seek medical attention. She attended a local clinic, where she received intravenous nonsteroidal anti-inflammatory drugs (NSAIDs); however, the pain persisted with high intensity. About 12 hours after the chiropractic session, she developed persistent left lower limb weakness. Functional limitation required the use of a walker for ambulation.

**Figure 1:**
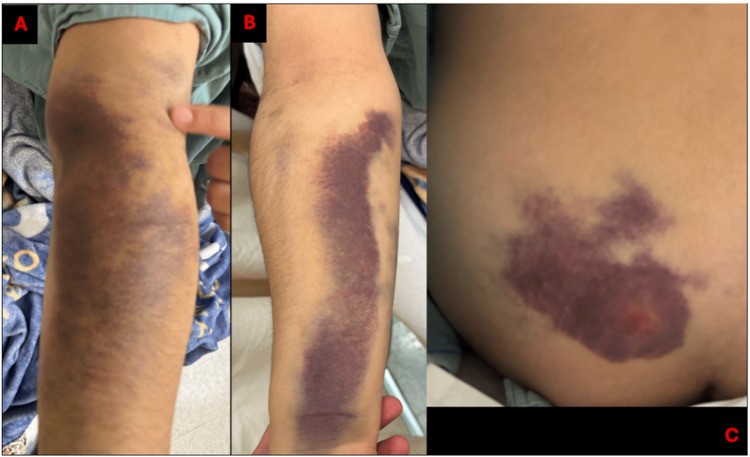
Clinical images of the patient Multiple ecchymoses were observed following chiropractic manipulation. (A) Dorsum of the right forearm; (B) Ventral aspect of the right forearm; (C) Sacral area.

On physical examination, the patient presented with large ecchymoses on the right forearm and sacral region (Figure 1).

Neurological examination findings were as follows: Right upper limb: strength 5/5 according to Daniel’s scale in C6-T1 distribution, preserved sensitivity in corresponding dermatomes, full range of motion, capillary refill <2 seconds, no distal neurovascular compromise, and ecchymoses on the dorsal and ventral forearm. Left upper limb: strength 5/5 according to Daniel’s scale in C6-T1 distribution, preserved sensitivity, full range of motion, capillary refill <2 seconds, no distal neurovascular compromise. Left lower limb: inability to flex the hip or extend the knee; strength 2/5 in L2-L4 distribution according to Daniel’s scale; sensitivity reduced by 50% compared to the contralateral limb in L2-S1 dermatomes; passive and active range of motion limited due to pain; capillary refill <2 seconds; no signs of distal vascular compromise. Right lower limb: strength 5/5 according to Daniel’s scale, sensitivity preserved in L2-S1 dermatomes, full range of motion, capillary refill <2 seconds, no distal neurovascular compromise.

At presentation, she was ambulatory with a walker, with no active bleeding, though multiple large ecchymoses were noted.

Diagnostic assessment

Initial evaluation included anteroposterior and lateral radiographs of the lumbar spine, a computed tomography (CT) scan, and a non-contrast magnetic resonance (MRI).

Radiographs revealed no cortical discontinuity, normal spinopelvic parameters, adequate bone mineralization, no vertebral displacement, and normal foraminal space.

**Figure 2:**
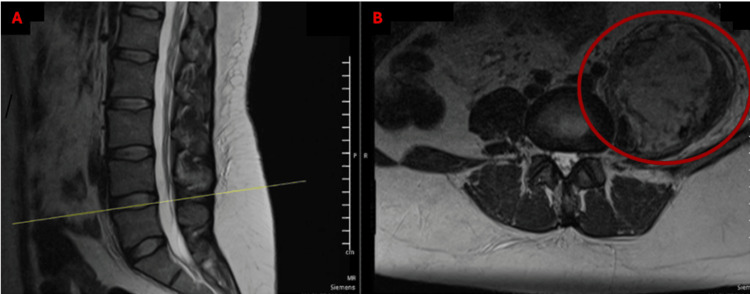
Initial iliopsoas hematoma identified on magnetic resonance imaging (MRI) Simple MRI obtained during admission in a T2 sequence, showing an intraperitoneal collection in the psoas muscle of approximately 896 cc. (A) Sagittal section; (B) Cross-section Cc: cubic centimeters

**Figure 3:**
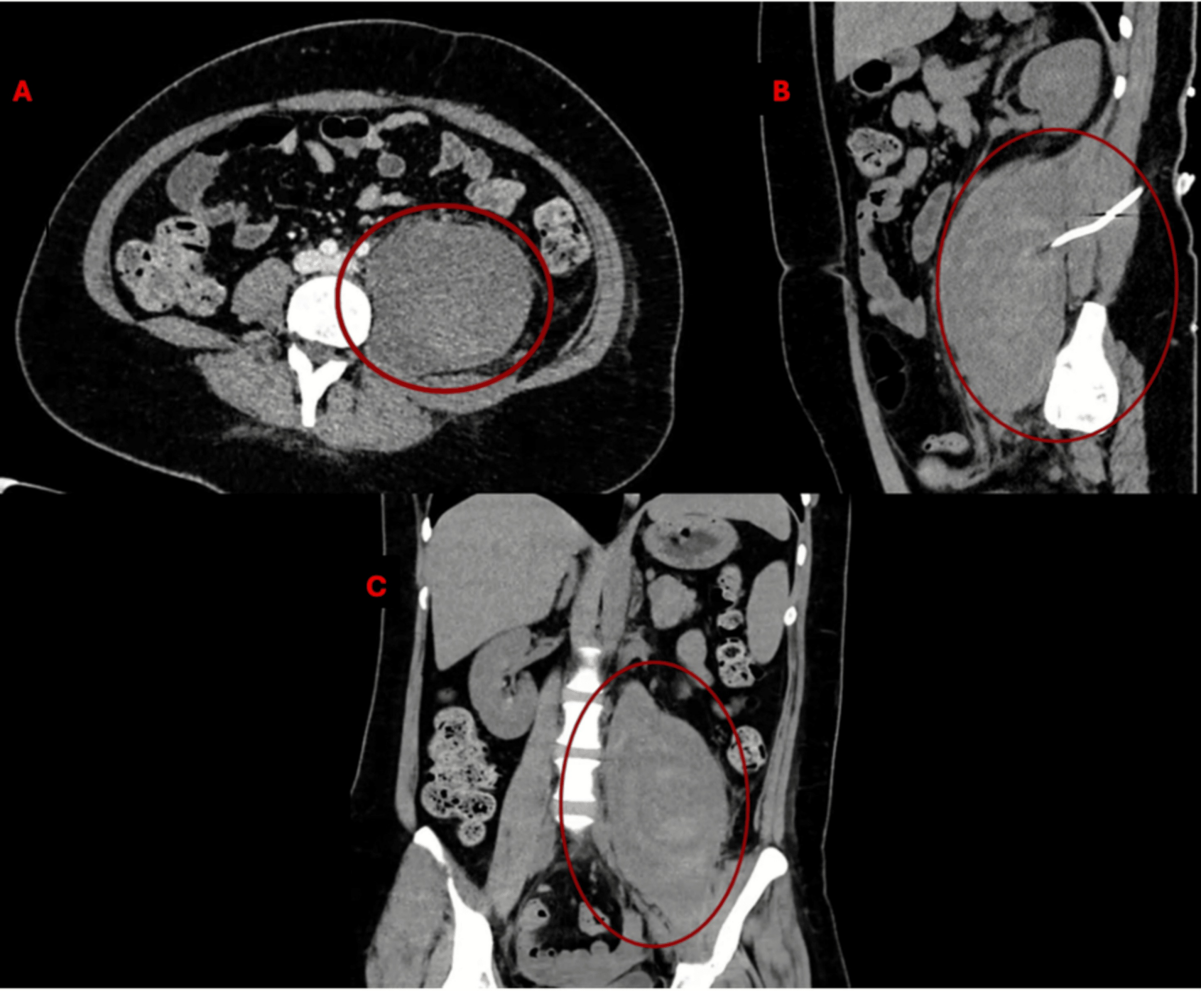
Hematoma in the iliopsoas noted via initial contrast-enhanced computed tomography (CT) scan Cross-section (A) and sagittal section (B) demonstrating correct placement of drainage via radiointervention. Pane C showcases the coronal section.

A CT scan and MRI demonstrated a large retroperitoneal lesion suggestive of a hematoma. Further evaluation with a contrast-enhanced CT scan confirmed the presence of a heterogeneous, irregular, poorly defined soft tissue lesion in the left iliopsoas muscle, containing hyperdense areas and gas locules, measuring 13.2 × 6.6 × 5.7 cm, with an estimated volume of 896 cc, consistent with hematoma (Figures 2-3). A drainage catheter was noted in an adequate position. Adjacent reactive lymphadenopathy was observed. No free intraperitoneal fluid was present.

Therapeutic intervention

The patient was admitted to the hospital for pain control with intravenous weak opioids. Hemostatic management was initiated with antihemophilic factor VIII/VWF concentrate, 2500 U intravenously every 24 hours for five days, repeated every three months.

**Figure 4:**
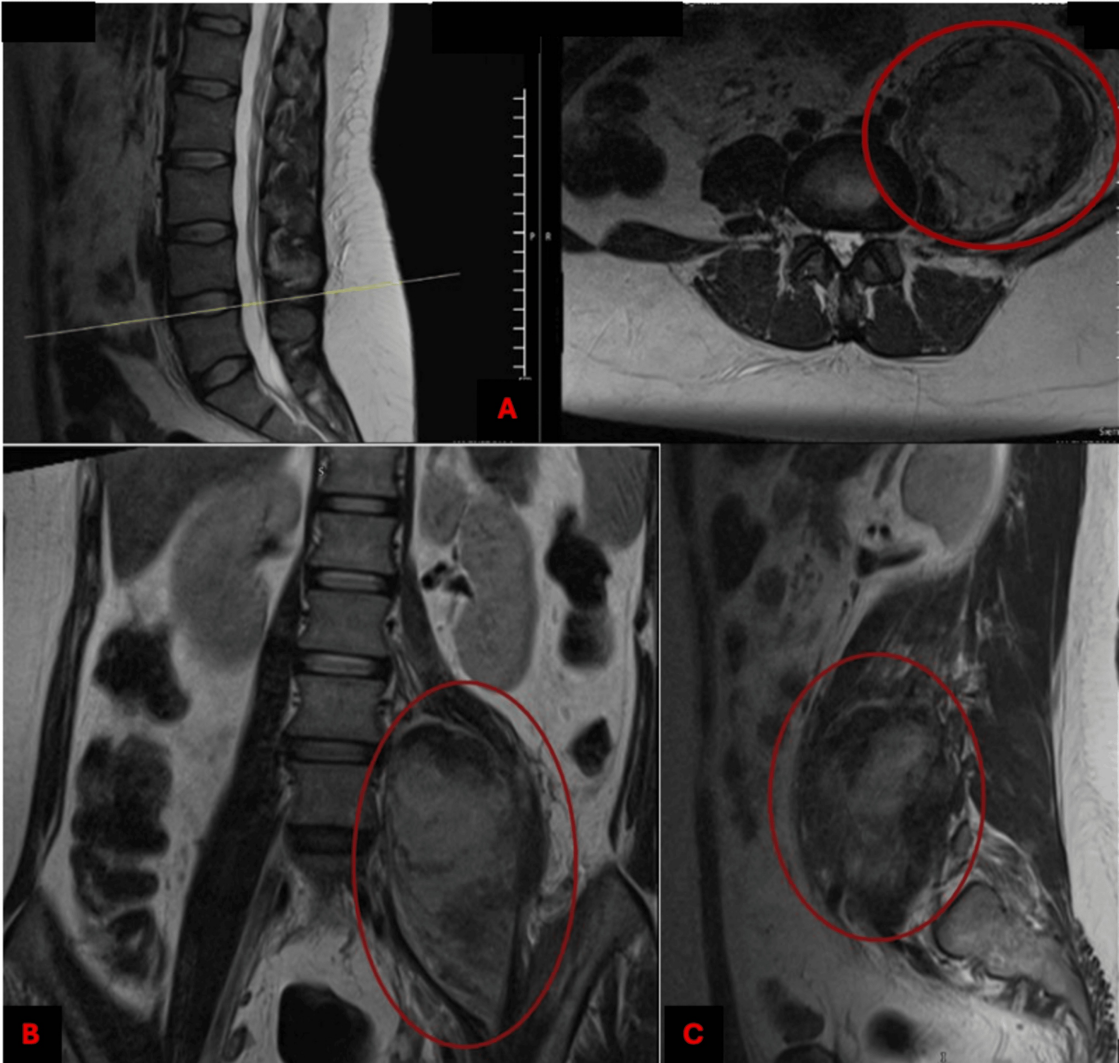
Hematoma in the iliopsoas visualized via magnetic resonance imaging (MRI) after drainage at the one-month follow up. Contrast MRI in a T2 sequence showing a decrease in the initial volume to 352 cc. (A) Cross-section; (B) Coronal section; (C) Sagittal section. Cc: cubic centimeters

**Figure 5:**
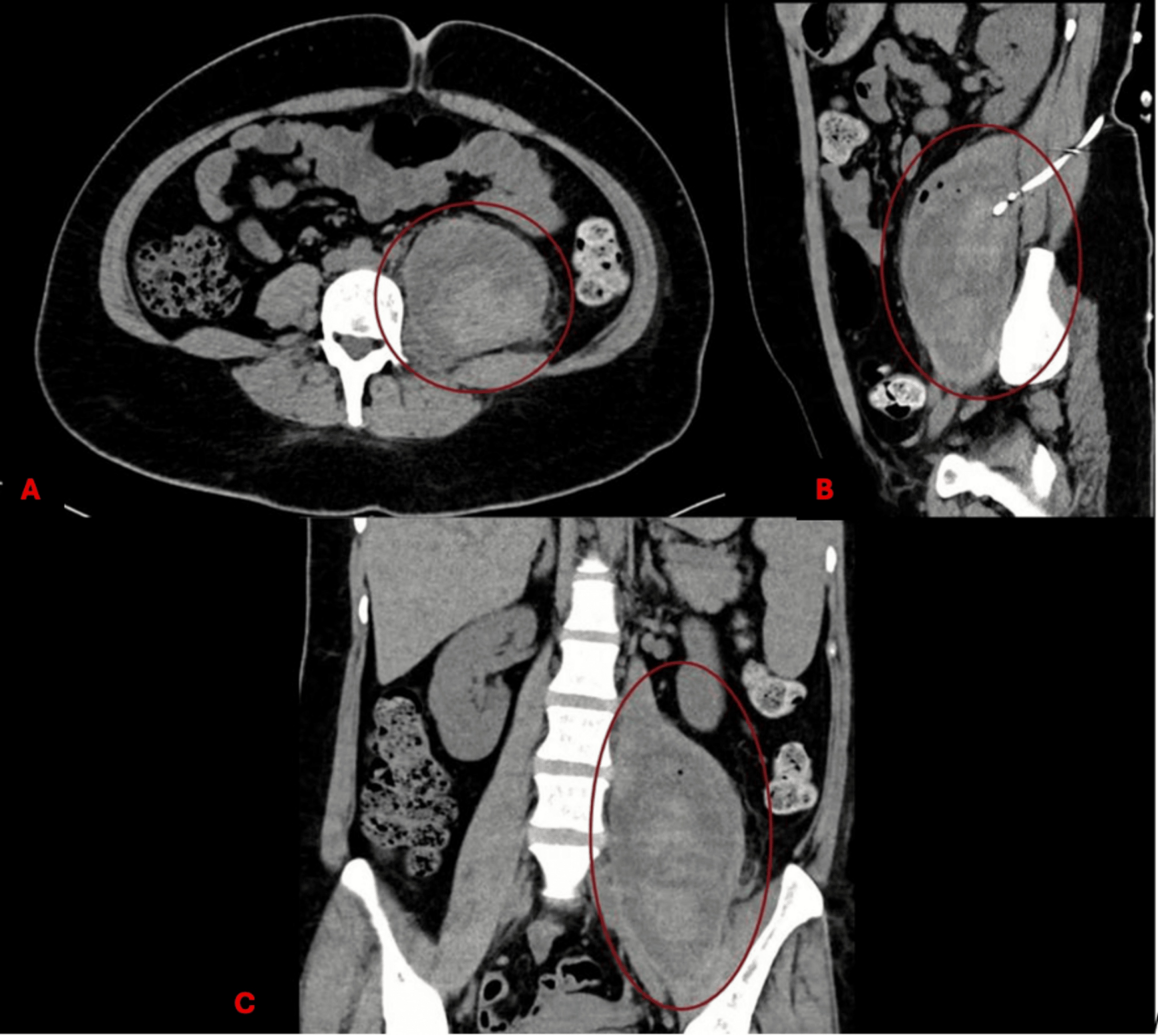
Hematoma in the iliopsoas vizualized via a contrast-enhanced computed tomography (CT) scan in the bone window at the one-month follow up. The CT scan shows a decrease in the initial volume to 352 cc. Cross-section (A) and sagittal section (B) show the correct placement of drainage in the hematoma. Pane C showcases the coronal section. Cc: cubic centimeters

**Figure 6:**
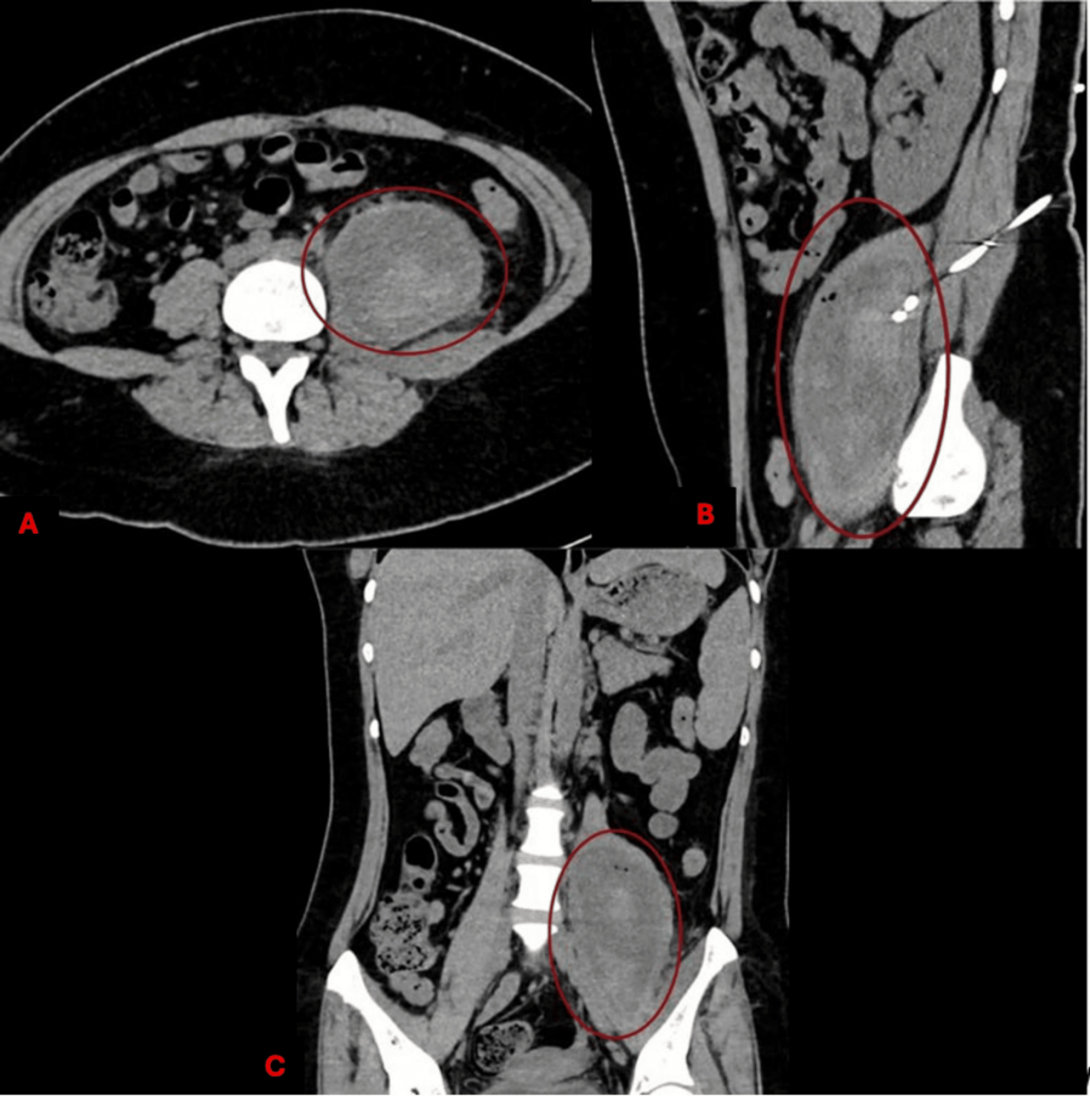
Hematoma in the iliopsoas noticed via contrast-enhanced computed tomography (CT) in a bone window during the three-month follow up. The CT scan shows a decrease in the initial volume to 257 cc. Cross-section (A) and sagittal section (B) show the correct placement of drainage in the hematoma. Pane C showcases the coronal section. Cc: cubic centimeters

The iliopsoas hematoma was drained by the interventional radiology team. A follow-up CT scan showed a residual irregular hypodense lesion (41 HU vs. 51 HU in contralateral muscle), measuring 10.2 × 7.5 × 8.8 cm, with an approximate volume of 352 cc, confirming a significant reduction compared to the previous study (896 cc to 352 cc). The drainage catheter was correctly positioned (Figures 4-5). After three months of follow-up, we noticed a decrease in the hematoma to 257 cc (Figure 6).

Follow-up

Following hematoma drainage, pain symptoms improved, and the patient began performing active hip, knee, and ankle flexion-extension movements, with some limitation against resistance.

Given her clinical improvement, the patient was discharged with outpatient follow-up in the spine surgery department.

At the six-month patient follow-up in the spine surgery clinic, she reported left lower limb paresthesias with significant improvement in pain of 3/10 on the Visual Analog Scale. On physical examination, there was partial recovery of motor function in the left lower limb (4/5 on Daniel’s scale), but asymmetry was observed, with reduced muscle mass compared to the contralateral side.

**Figure 7:**
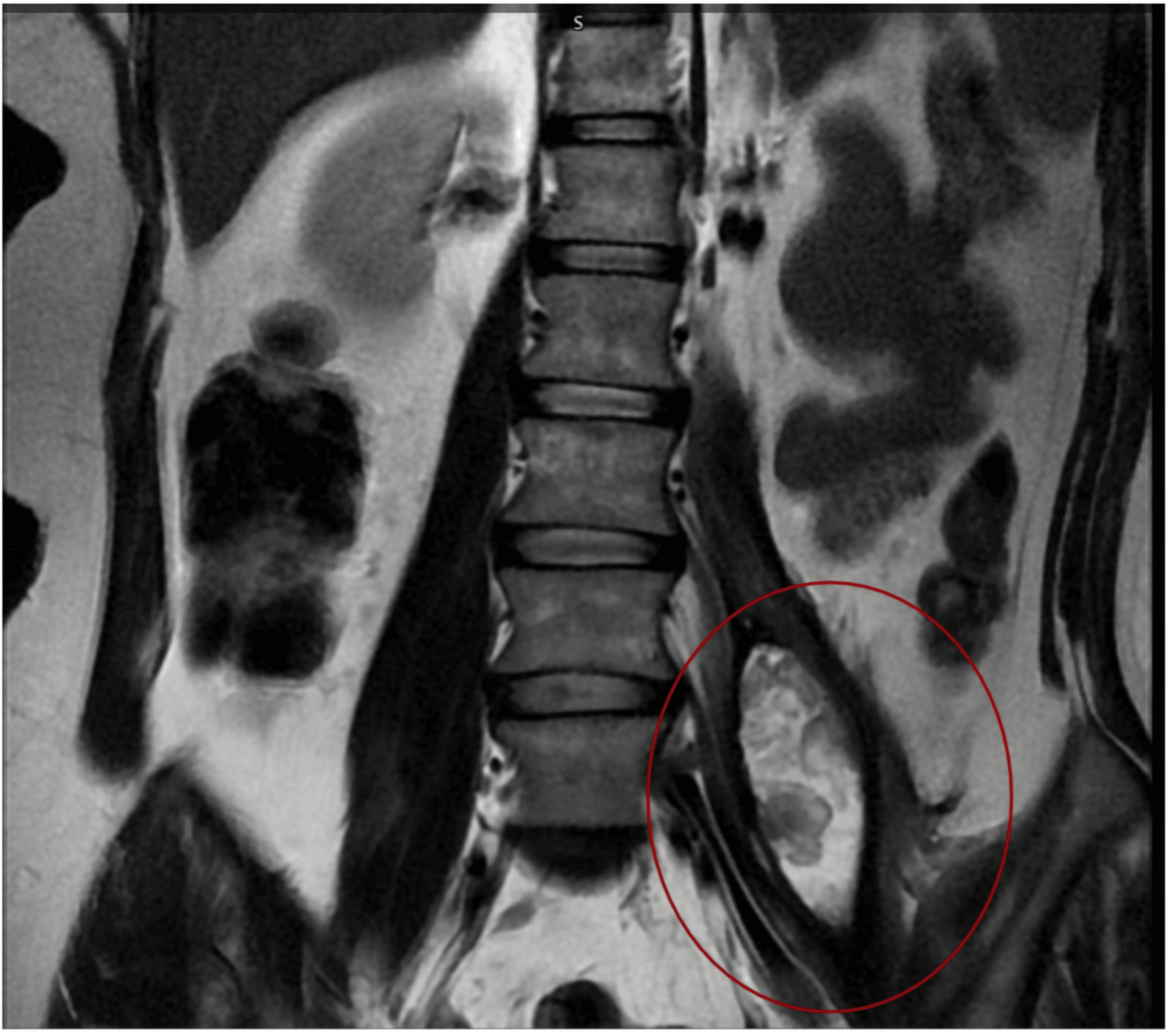
Iliopsoas hematoma diagnosed by a lumber contrast magnetic resonance imaging (MRI) in a T2 sequence during the six-month follow-up Coronal section shows a residual volume of 43 cc with heterogeneous content demonstrating calcification characteristics; no alteration is observed in adjacent abdominal or spinal structures. Cc: cubic centimeters

MRI revealed a residual 43 cc collection in the left psoas muscle with heterogeneous characteristics, suggestive of calcification of residual hematoma, associated with a left L3-L4 disc protrusion (Figure 7). Nerve conduction studies and electromyography demonstrated evidence of reinnervation in the left peroneal nerve. 

**Figure 8:**
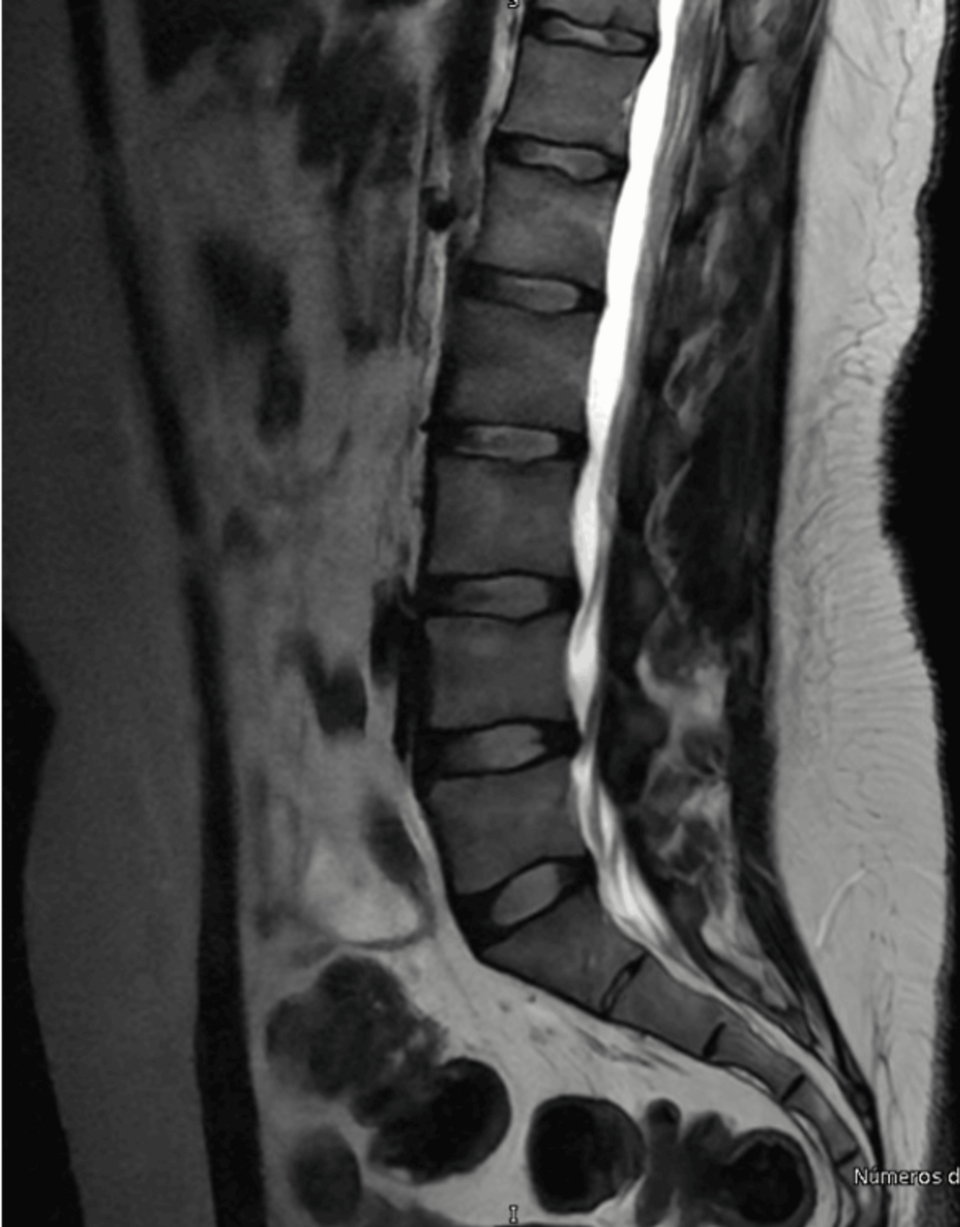
Degenerative changes noted in the lumbar spine via contrast magnetic resonance imaging (MRI) in a T2 sequence during the six-month follow-up. Sagittal section shows a L4-L5 disc protrusion (arrows). Adjacent intervertebral discs are observed without evidence of dehydration or significant degenerative changes. In the lower region, fat infiltration of paraspinal muscles is observed.

MRI showed degenerative changes in the lumbar spine, demonstrating a protrusion at the L4-L5 level, with no significant evidence of spinal cord involvement and no evidence of involvement of adjacent structures (Figure 8).

Physical rehabilitation focused on gait re-education and strengthening of the anterior thigh compartment. At this time, no surgical intervention was indicated, and the patient continued to be under follow-up to monitor muscular strength and distal neuropathy.

Following hematoma drainage, pain symptoms improved, and the patient began performing active hip, knee, and ankle flexion-extension movements, with some limitation against resistance.

## Discussion

This case illustrates the risks of chiropractic manipulation in patients with inherited bleeding disorders such as VWD. The patient developed a large iliopsoas hematoma with secondary femoral neuropathy, resulting in significant motor and sensory deficits.

Retroperitoneal and iliopsoas hematomas are recognized complications in coagulopathic patients and can lead to compression of the femoral nerve [6-10], presenting clinically with hip flexion and knee extension weakness, as well as sensory impairment in the anterior thigh. 

Management of these cases [10,11] involves pain control, correction of the underlying coagulopathy with factor replacement, and, in selected cases, interventional drainage. Early recognition is crucial to avoid long-term neurological sequelae.

The use of NSAIDs (such as ketorolac in this patient) at a non-specialized clinic complicates management, as these drugs exacerbate bleeding by inhibiting platelet function. [4,7,10] Multidisciplinary care involving hematology, interventional radiology, neurology, and rehabilitation is essential to achieve favorable outcomes.

In most case reports, during our research with this type of complication, patients describe intense refractory pain to analgesics and weak opioids, needing to resort to high-intensity opioids subcutaneously or intravenously (morphine, fentanyl) [10-13].

Chan et al. [5] reported that the majority of psoas hematomas can be absorbed spontaneously and do not require any further intervention measures, as opposed to open surgery, as it can further aggravate bleeding.

There is controversy regarding when to treat a psoas hematoma conservatively or surgically. Information in prior studies regarding the hemodynamic instability and complete palsy caused by the size of the hematoma could be a clear indication for a surgical procedure [5-7]. 

In most of the reported cases, they do not establish the specific volume of each of the hematomas; however, they establish the measurements of the same based on the CT scan or the MRI, so in our study, we performed the calculation based on a validated and established formula to calculate volumes in various cavities, such as the brain or abdomen. 

The ABC/2 formula is a simple method used to estimate hemorrhage volume on CT imaging. It is based on the assumption that the hemorrhage approximates an ellipsoid shape.

Measurement A corresponds to the largest hemorrhage diameter on the CT slice with the greatest area, B is the diameter perpendicular to A on the same slice, and C represents the approximate depth of the hemorrhage, calculated as the number of CT slices containing hemorrhage multiplied by slice thickness. The product of these three measurements divided by two provides an accurate estimation of hemorrhage volume, allowing rapid assessment with high correlation to computer-assisted planimetric techniques [8].

The use of this equation to calculate volumes is currently in force. The calculation of the volume that we established is shown in Table 1. 

**Table 1:** Reported cases of iliopsoas hematoma, diagnosis, and management MRI: magnetic resonance imaging; CT: computed tomography; VTE: venous thromboembolism. This table shows the management in each of the case reports of hematoma in the iliopsoas regardless of their etiology. The unreported volumes are due to the fact that no hematoma dimensions are reported in the published literature.

Author/year	Age	Complication	Cause	Confirmation/Diagnosis	Volume (cm3)	Management/Treatment
Kawarat et al. [9]	40 years	Psoas hematoma and abscess	Pentrating trauma	CT scan.	119.2	Antibiotics and ultrasound-guided drainage
Zhao et al. [14]	20 years	Spontaneous iliopsoas hematoma	No trauma	CT scan	Not reported	Factor replacement and physiotherapy
Alsadery et al. [15]	69 years	Psoas hematoma	Lumbar fusion surgery (L3-L5)	MRI, CT scan	102.5	Conservative treatment
Canelles et al. [16]	1. 34 years; 2. 84 years; 3. 80 years	1. Iliopsoas hematoma; 2. Iliopsoas hematoma; 3. Iliopsoas hematoma	1. Anticoagulant treatment after hip intervention; 2. Anticoagulant treatment after VTE	1. CT scan; 2. CT scan; 3. Ultrasound	1. Not reported; 2. 478; 3. Not reported	1. Analgesia and absolute rest; 2. Vitamin K and procoagulant factors; 3. Vitamin K and rest.
Pirouzmand et al. [17]	15 years	Iliopsoas	Hip extension injury when falling backward on ice while skating.	CT scan	Not reported	Conservative treatment

Currently, there is no specific hematoma volume that universally indicates surgery. However, based on our research, we identified reported volumes and compiled a table (Table 1), summarizing hematoma size and the management approach described by each author in their respective cases.

These are not parameters for a specific therapy or indication between a conservative or surgical procedure regarding the hemodynamic stability or the palsy, but we illustrate that there may be a correlation between the volume of the psoas hematoma and the treatment.

Although iliopsoas muscle hematomas have been described through external trauma, such as surgery, or as an intrinsic consequence of coagulopathies, such as VWD, there is no evidence of hematoma in the iliopsoas muscle as a consequence of joint manipulation that has been reported [9,10,11]. 

There are about 1,00,000 certified chiropractors worldwide, where health professionals receive formal training in chiropractic and make it a safe practice; however, there are false professionals in chiropractic who manipulate patients with various conditions and damage their integrity, so it is recommended to go to a health professional suitable and certified for any manipulation at any vertebral level. In addition, for health professionals dedicated to this clinical practice, there are guides to improve joint manipulation techniques, particularly spinal manipulation, which establish techniques, recommendations, and follow-up for the patient. Patients with inherited bleeding disorders should be counseled on the risks of alternative therapies such as chiropractic manipulation. Iliopsoas hematoma with femoral neuropathy represents a potentially severe but preventable complication. 

## Conclusions

VWD predisposes patients to severe bleeding complications even after minor trauma or manipulative therapies. This case emphasizes the importance of avoiding high-risk procedures such as chiropractic manipulation in coagulopathic patients, the need for early diagnostic workup when neurological deficits arise, and the benefit of coordinated multidisciplinary management. Despite the use of chiropractic as an alternative medicine, it is important to recognize the possible consequences of its use in clinical practice and to weigh the risk/benefit of using this therapy, since sometimes it can have irreversible sequelae.
